# A longitudinal analysis of soil salinity changes using remotely sensed imageries

**DOI:** 10.1038/s41598-024-60033-6

**Published:** 2024-05-06

**Authors:** Soraya Bandak, Seyed Alireza Movahedi-Naeini, Saeed Mehri, Aynaz Lotfata

**Affiliations:** 1 Department of Soil Sciences, Gorgan University of Agricultural Sciences and Natural Resources, Gorgan, Iran; 2 Department of Geospatial Information Systems, Faculty of Geodesy and Geomatics Engineering, K. N. Toosi University of Technology, Tehran, Iran; 3 Department of Pathology, Microbiology, and Immunology, School Of Veterinary Medicine, University of California, Davis, USA

**Keywords:** Soil salinization, Remote sensing, Predictive modeling, Decision tree, Environmental impact, Scientific data, Electronic properties and materials, Agroecology

## Abstract

Soil salinization threatens agricultural productivity, leading to desertification and land degradation. Given the challenges of conducting labor-intensive and expensive field studies and laboratory analyses on a large scale, recent efforts have focused on leveraging remote sensing techniques to study soil salinity. This study assesses the importance of soil salinity indices’ derived from remotely sensed imagery. Indices derived from Landsat 8 (L8) and Sentinel 2 (S2) imagery are used in Random Forest (RF), eXtreme Gradient Boosting (XGBoost), Decision Tree (DT), and Support Vector Machine (SVR) are associated with the electrical (EC) conductivity of 280 soil samples across 24,000 hectares in Northeast Iran. The results indicated that the DT is the best-performing method (RMSE = 12.25, MAE = 2.15, R^2^ = 0.85 using L8 data and RMSE = 10.9, MAE = 2.12, and R^2^ = 0.86 using S2 data). Also, the results showed that Multi-resolution Valley Bottom Flatness (MrVBF), moisture index, Topographic Wetness Index (TWI), and Topographic Position Indicator (TPI) are the most important salinity indices. Subsequently, a time series analysis indicated a reduction in salinity and sodium levels in regions with installed drainage networks, underscoring the effectiveness of the drainage system. These findings can assist decision-making about land use and conservation efforts, particularly in regions with high soil salinity.

## Introduction

Soil salinization profoundly affects soil productivity, nutrient availability, and plant physiology and biochemistry, especially in arid and semi-arid regions. Intensive irrigation in these areas brings saline groundwater to the surface, leading to overflow zones where evaporite minerals precipitate^1–3^. The salinity adversely affects crop water and fertilizer uptake and soil fertility enhancement, impacting more than 20% of the world's irrigated land^4^.

Soil Electrical Conductivity (EC) is a key indicator of soil salinity measurements, strongly correlating with salinity levels^5^. Mapping EC can enhance understanding of soil genesis processes in arid and semi-arid soils and aid agricultural management. However, due to its complex spatial variation, soil EC estimation is more challenging than other soil properties (e.g., soil organic carbon), necessitating the development of more reliable methods.

Remotely sensed images, including Landsat 8 (L8) and Sentinel 2 (S2), are extensively used for salinity analysis, offering spectral diversity and cost-effectiveness in soil salinity mapping^6–10^. Within this context, Erkin et al.^11^ used L8 for temporal soil salinity analysis in Kashgar. They found that due to the continuous increase in inland reclamation and insufficient drainage, salinized arable land has steadily increased, and the average salinity of the cropland reached higher than 5.1 g per kilogram of soil. Taghizadeh-Mehrjardi et al.^12^ identified that the S2 satellite image is a suitable and cost-effective data source for soil salinity assessment due to its short revisit interval, multiple spectral bands, and high spatial resolution. Wu et al.^16^ used Landsat 5 TM and ALOS L-band radar to create a soil salinity map in the Mussaib region of Central Mesopotamia, finding that the Random Forest (RF) algorithm outperformed Support Vector Regression (SVR). Wang et al.^13^ compared L8 and S2 images in the Ebinur Lake wetland using the cubist model, finding that the S2 image is superior for salinity estimation. Wang et al.^14^ have shown that the Cubist model with L8 image is superior for salinity estimation compared to using S2 with that model in Ebinur Lake Wetland National Nature Reserve, China. Wang et al.^15^ demonstrate the effectiveness of S2 images in distinguishing between saline and non-saline areas using the RF algorithm. Their study also highlights the capability to monitor changes in soil salinity levels between dry and wet seasons using remotely sensed images by generating region-specific maps.

Ma et al.^16^ digitally mapped salinity distribution in the Werigan-Kuqa oasis, analyzing the evolution characteristics and driving factors using a machine learning approach and field data. The eXtreme Gradient Boosting (XGBoost) model significantly enhances prediction accuracy and salinity mapping, illustrating spatial and temporal changes over 25 years. Ge et al.^17^ introduced a hybrid machine learning framework utilizing S2 image and environmental determinants, achieving a notable improvement in soil salinity mapping accuracy by 5–8%. These studies show that the L8 satellite image is more effective for monitoring soil salinity than the S2 image^13,18–20^. Also, researchers have employed RF^21^, XGBoost^22^, Decision Tree (DT)^23^, and SVR^24,25^ as predictive tools in their investigations, and these models consistently demonstrated robust and reliable performance in the context of soil salinity prediction.

This study's main aim is to analyze soil salinity variations in a drainage area using L8 and S2 imagery data alongside machine learning methods. Additionally, it compares salinity levels before and after installing a regional drainage network.

## Materials and methods

**Figure 1:**
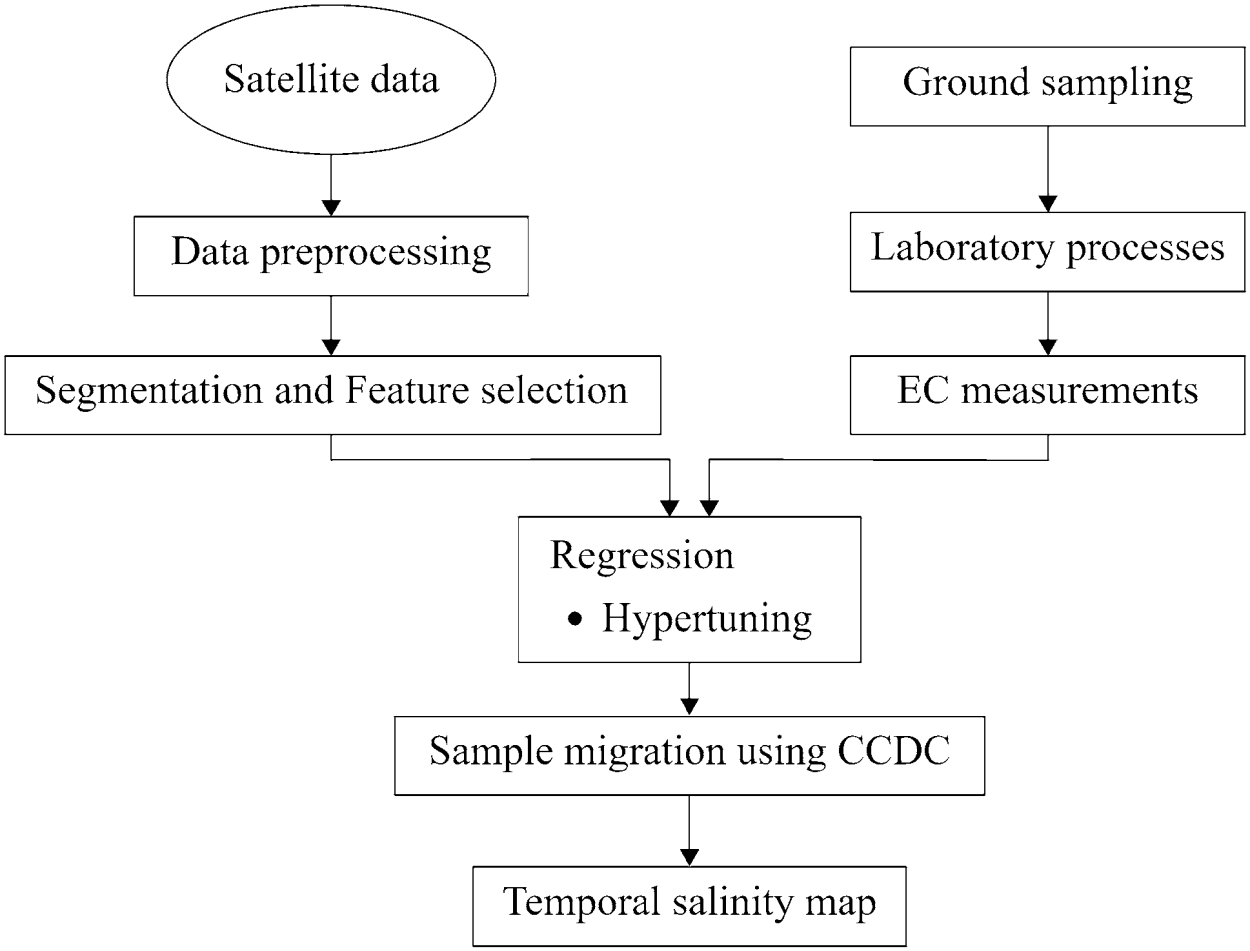
Workflow of the study.

Figure 1 provides a summary of the workflow in this study. The initial step involves preprocessing the imagery data for image segmentation delineating areas for collecting soil sampling field data. The second step entails collecting field soil sample data to measure EC as an indicator of soil salinity. Following this, preprocessed S2 and L8 imagery data is utilized to extract indices, which are then used for feature selection using machine learning algorithms.

The measured EC of soil samples is used as a dependent variable for predicting soil salinity, while the independent variables are derived from S2 and L8 imagery data. Considering that indices are extracted at the object-level segments rather than the pixel level. An object-based feature extraction strategy simplifies analysis, reduces noise, and enhances accuracy^26–28^. Also, this study uses a hyperparameter tuning evaluation in regression models to optimize model performance and identify the most suitable parameters. Subsequently, sample migration is performed based on the selected most informative features to conduct a time-series analysis using the Continuous Change Detection and Classification (CCDC) algorithm^29^. Finally, the paper creates a time series of EC maps to analyze temporal variations of soil salinity over time. These maps give a better understanding of the EC patterns and trends over time.

### Study area

**Figure 2:**
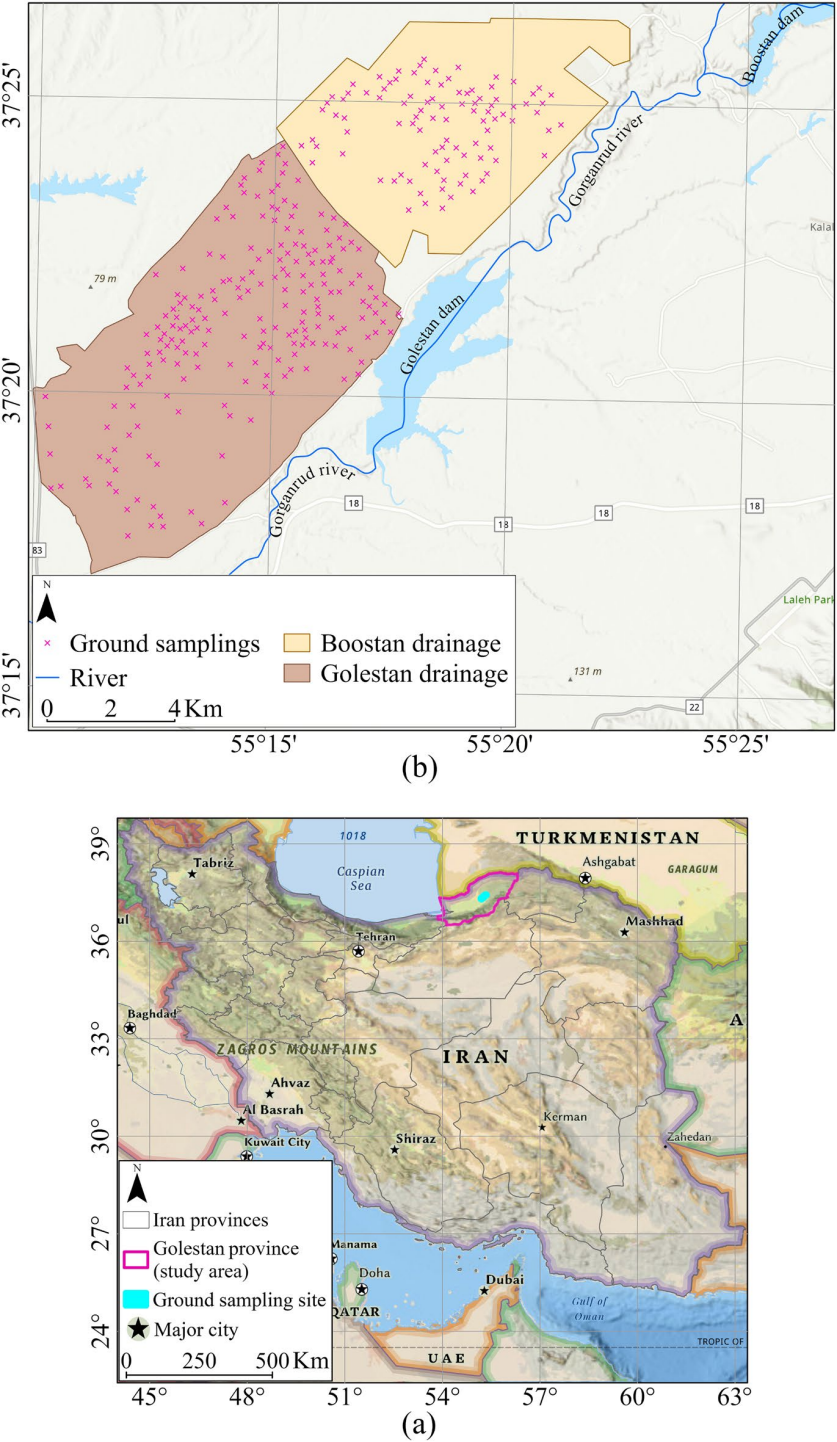
Location of Golestan province and ground sampling site (**a**). overlaying on the National Geographic Style Map in Esri ArcGIS (version 10.8)^32^, Boostan and Golestan drainage area, and ground sampling locations (**b**). overlaid on the World Hillshade base map in Esri ArcGIS (version 10.8)^32^.

Within the geographical coordinates ranging from 55° 10′ to 55° 22′ East longitude and 37° 15′ to 37° 25′ North latitude, the study area was conducted in the Gonbad region of Golestan Province, situated in the northern part of Iran (Fig. 2a). This region experiences a temperate climate and features predominantly flat terrain. Over the past two decades, it has received an average annual rainfall of 455 mm, with a mean annual temperature of 17 °C^30^. Notably, the yearly minimum temperature recorded in this period was 15.6 °C, while the maximum temperature reached 37.5 °C. Furthermore, based on the soil taxonomy system established by the United States, the soil type in this area is classified as a typical Haploxerept^31^.

The study area, located on the right flank of the Gorganroud River, spans approximately 24,000 hectares of farmlands in the Golestan and Boostan drainage networks (Fig. 2b), which are irrigated from the Golestan and Boostan dams, respectively. Soil salinity is a prevalent issue in the Gonbad Kavus area, attributed to high groundwater levels and subsequent water evaporation, leaving behind salts in the soil. Therefore, this study aimed to assess the potential of optical Earth observation imagery for predicting soil surface salinity in the eastern region of Gonbad City.

### Data

#### Image segmentaion

We utilized S2 imagery for land segmentation, which was employed for field sampling and extraction of indices within each segment. The S2 multispectral imagery comprises 13 bands and has a spatial resolution of 10 m in visible bands and 20 m in the remaining. The S2 dataset, processed by ESA^33^, is also accessed through the Google Earth Engine (GEE)^34^. Utilizing S2 imagery data, we grouped nearby pixels based on similar characteristics such as color and texture^39^. We employed the K-Nearest Neighbors (KNN) algorithm to group nearby pixels or image segmentation. We tried different grouping levels to find the best one, considering things like how clear the images were. We used various maps and data layers, like contour maps and soil maps, to help with this process. Then, we looked for soil samples in each segmented region (see Supplementary A for detailed information on the segmentation process).

#### Sampled soil data as the dependent variable

On July 1, 2020, during the summer season, a field survey and soil sample collection were carried out to ensure compatibility with imagery from the S2 and L8 satellites. Global Positioning System (GPS) receivers were employed to pinpoint the sampling locations. Each sampling point was established as a circle with a 5-m radius, from which eight individual soil samples were extracted from the 0–10 cm depth and subsequently combined into a single composite sample. All soil samples were transported to the laboratory for further analysis to determine their moisture content and conductivity. First, 200 g of fresh soil samples were weighed and placed in a drying apparatus. Subsequently, 20.00 g from each naturally air-dried soil sample were precisely measured to prepare a soil extract with saturated paste extracts. The EC was then calculated using the saturation extract method.

#### S2 and L8 imagery data were used to extract indices as independent variables

The independent variables used in this study are sourced from L8 and S2 imagery data. The L8 is a multispectral satellite with a spatial resolution of 30 m. It is provided by the United States Geological Survey (USGS)^35^ and accessed through GEE^35^. To select the most suitable images, we filtered image collections available between April and July 2020 to exclude images having more than 20 percent cloud cover. The filtering process allows the creation of a composite image with no cloud cover.

Twenty-six environmental variables (including auxiliary variables) are used for soil salinity estimation. These variables are used in feature selection to identify the most important features in soil salinity estimation (see Supplementary A for detailed information on feature selection). Table 1 provides a detailed overview of these variables (indices), including their formulas.

**Table 1:** Variables used in predicting soil salinity.

Covariates	Definition	Reference
Elevation^36^	Height above sea level (m)	DEM
Diffuse Insolation^37^	Calculate the diffuse incoming solar radiation	DEM
Topographic Position Index (TPI)	Calculate with SAGA	DEM
Mean Annual Precipitation (MAP)^38^	Derived from the monthly rainfall values	WordClim
Mean Annual Temperature (MAT)^38^	Derived from the monthly temperature values	WordClim
Normalized Difference Salinity Index (NDSI)^39^	$\frac{(Red-NIR)}{(NIR+Red)}$	L8/S2
Vegetation soil salinity index (VSSI)^40^	$2\times GREEN-5\times (RED+NIR)$	L8/S2
Soil Adjusted Vegetation Index (SAVI)^41^	$\frac{(1+L)(NIR-Red)}{(NIR+Red+L)}$	L8/S2
Normalized Difference Vegetation Index (NDVI)^42^	$\frac{(NIR-Red)}{(NIR+Red)}$	L8/S2
Enhanced Vegetation Index (EVI)^43^	$\frac{2.5(NIR-Red)}{\left(NIR+6R-7.5B\right)+1}$	L8/S2
Generalized Difference Vegetation Index (GDVI)^44^	$\frac{({NIR}^{2}-{RED}^{2})}{({NIR}^{2}+{RED}^{2})}$	L8/S2
Normalized Difference Water Index (NDWI)^45^	$\frac{(NIR-SWIR)}{(NIR+SWIR)}$	L8/S2
Modified Normalized Difference Water Index (MNDWI)^46^	$\frac{(GREEN-MIR)}{(GREEN+MIR)}$	L8/S2
Normalized Difference Salinity Index (NDSI)^39^	$\frac{(RED-NIR)}{(RED+NIR)}$	L8/S2
Multi-resolution Valley Bottom Flatness (MrVBF)^47^	DEM	DEM
Topographic Wetness Index (TWI)^48^	$ln(\frac{local upslope}{slope})$	DEM
‌ Brightness Index (BI)^39^	$\sqrt{({{\text{Green}})}^{2}+{({\text{RED}})}^{2}}$	L8/S2
Salinity 1(S1)^39^	$\sqrt{({\text{Green}}\times {\text{Red}})}$	L8/S2
Salinity 2 (S2)^49^	$\sqrt{({\text{Blue}}\times {\text{Red}})}$	L8/S2
Salinity 3 (S3)^50^	$\frac{({\text{Red}}\times {\text{Green}})}{{\text{Blue}}}$	L8/S2
Salinity 4 (S4)^50^	$\frac{{\text{Blue}}}{{\text{Red}}}$	L8/S2
Extended NDVI^13^	$\frac{(\text{NIR }+\text{ SWIR}2 - \text{ Red}) }{(\text{NIR}+\text{SWIR}2 +\text{Red})}$	L8/S2
Extended EVI^13^	$\frac{2.5*({\text{NIR}}+\text{ SWIR}1-{\text{Red}}) }{\text{ NIR}+{\text{SWIR}}1+6*{\text{Red}}-7.5*{\text{Blue}}+1}$	L8/S2
Canopy Response Salinity Index^51^	$\sqrt{\frac{([({\text{NIR}}\times {\text{Red}})- ({\text{Green}}\times {\text{Blue}})] }{[({\text{NIR}}\times {\text{RED}})+ ({\text{Green}}\times {\text{Blue}})])}}$	L8/S2
Two-band EVI^52^	$2.5\times \frac{(\text{NIR} - \text{ Red})}{(NIR+2.4\times RED+1)}$	L8/S2
Difference Infrared Index (NDII)^53^	$\frac{(NIR-\text{SWIR }1)}{({\text{NIR}}+\text{SWIR }1)}$	L8/S2

#### Machine learning regression analysis

Regression analysis is a commonly used statistical tool to study relationships between factors, making it straightforward to analyze multifactor data^54^. This study employs SVR^44,55–59^, RF^22,58, 60–62^, DT^63,64^, and the XGBoost^16,65–68^ methods to investigate the association between remotely sensed imagery data and field soil salinity. The Scikit-learn for Python (version 1.3.0) is used to implement these algorithms^69^.

#### Regression and hyperparameter tuning for feature selection

Hyperparameter tuning is essential for maximizing the performance of machine learning algorithms^70,71^. Algorithms, such as DT, RF, XGBoost, and SVR, involve various types of hyperparameters, and the fine-tuning of these parameters directly influences the algorithm's effectiveness^72^. Several methods can be utilized to optimize parameters and enhance the performance of models, such as the local search method^73^. This paper uses the BayesSearchCV and GridSearchCV methods to fine-tune the hyperparameters of RF, XGBoost, DT, and SVR models using the scikit-optimize library (version 0.8.1) in Python^74^. Bayesian search CV is grounded in Baye’s rule of conditional probability, using prior knowledge to calculate posterior probabilities. Detailed information on the hyperparameters is provided in supplementary B.

#### DT

A DT, one of the simplest yet most successful machine learning methods, uses a divide-and-conquer approach to classify and regress large databases^75,76^. This makes DT one of the common machine-learning methods^75^. A DT is a non-parametric supervised learning method with a tree-like structure^77^. It acts as a function taking input from a vector of attribute values and returning a “decision.” The decision is reached through a series of tests. Each internal node in the tree corresponds to an examination of the value of one of the input attributes, and the branches from that node are labeled with the attribute’s possible values. Each leaf node in the tree specifies a value to be returned by the function^76^. Both input and output values are discrete (classification) or continuous (regression). A regression tree has a linear function of some subset of numerical attributes at each leaf rather than a single value. The learning algorithm must decide when to stop splitting and begin applying linear regression over the attributes^54,76^. DTs are prone to overfitting, meaning they follow the peculiarities of the training dataset too closely and may not perform well on a new dataset, i.e., the test dataset. In such cases, the general predictive accuracy of overfitting DTs will be low, i.e., generalization accuracy^78^. One approach to improve the generalization accuracy is to construct multiple individual trees using a subset of the observations^78,79^, which is the main idea of the RF algorithm^80^. Detailed information on the DT hyperparameters are provided in Supplementary Table S1.

#### RF

The RF is an ensemble supervised learning algorithm using a collection of DTs for prediction. Individual trees are constructed by bootstrapping the dataset and averaging the results of all the trees to make the final prediction. Bootstrap aggregating or bagging helps reduce overfitting^78^. The RF can be used to classify categorical target variables and the regression of continuous variables^81^. For regression purposes, at each branching of the regression tree, the mean of the samples on the leaf nodes and the Root Mean Square Error (RMSE) formed between each sample are calculated. Following the minimum RMSE of leaf nodes as a branching condition, the regression tree stops when no more features are available, or the overall RMSE is optimal^77^. The key to creating an accurate model is ensuring that the base learner, typically a regression tree, is as uncorrelated as possible to produce a robust generalization ability^80^. Detailed RF hyperparameter information is provided in Supplementary Fig. S2 and Supplementary Table S2.

#### SVR

The Support Vector (SV) algorithm, a set of related supervised learning methods, was first introduced for pattern recognition^82^ and then generalized to solve regression problems^83^. The SVR is a tool for overall and short-term forecasting or when real-time analysis is required^84^. Also, it works in an infinite-dimensional space, giving it an edge over similar networks^84^. The SVR investigates the relationship between one or more predictor variables and a real-valued (continuous) dependent variable^85^. SVR finds a best-fitting hyperplane to data points in a continuous space, while in linear regression, a line is fitted to the data points. In SVR, the best-fitting hyperplane passes through as many sample points as possible within a certain distance, called a margin, which is defined to be the smallest distance between the decision boundary and any of the samples^86^. SVR is very sensitive to the input data type, as it can produce incorrect results if the data spans a wide range. Therefore, data normalization is an essential step in using SVR^84^. Detailed information on the SVR hyperparameters are provided in Supplementary Fig. S3.

#### XGBoost

The XGBoost algorithm was proposed by Chen and Guestrin^87^. A scalable implementation of XGBoost is robust and highly efficient^88,89^. It uses the Classification and Regression Trees (CART) and is jointly decided by multiple related DTs^90^. In this structure, the input sample of the next DT is related to the training and prediction results of the previous DT. Like most machine learning algorithms, in XGBoost, the objective is to minimize the sum of the loss function to control the accuracy and complexity of the mode^90^. Detailed information on the XGBoost hyperparameters are provided in Supplementary Table S3.

#### Migration sample with algorithm CCDC

The fundamental concept behind CCDC involves fitting a simple harmonic model to a cloud-free time series and detecting changes when the difference between observed and predicted pixel values surpasses a predefined threshold for consecutive periods. Notably, Chen et al.^87^ enhanced the CCDC algorithm by introducing a multi-harmonic model capable of fitting intricate phenological profiles in cultivated land. The paper used the CCDC method to select unchanged samples and generate additional field samples for each period during which field data were not collected in those years. These unchanged field samples serve as training and test data for all years. The process commenced with cloud masking of L8 images and the computation of two essential spectral indices: the NDWI and the NDSI (Table 1). Subsequently, these indices were leveraged with the CCDC model, as outlined in Eq. (1), to identify and isolate the unchanged field samples^91^.

1 $$F\left(i, x\right)={a}_{0,i}+{a}_{1,i}{\text{cos}} (2 \pi/{t}_{x}) +{b}_{1,i}{\text{sin}}(2 \pi/{t}_{x}) +{c}_{1,i}$$

where *i*, *x*, and *t* represent the spectral index, Julian date, and the number of days in a year (i.e., 365.25 days); *a*_*0,i*_ stands for the overall value of spectral index *i* of an L8 image; *a*_*1,i*_ and *b*_*1,i*_ specify the intra-year change. Furthermore, *c*_*1,i*_ pertains to interannual values, and these new samples estimate model values and residuals by comparing observed and modeled sample values, as outlined in Eq. (1). In this context, a threshold value of 20% was employed. If the residual exceeded this threshold, it was assumed that an interannual variation had occurred. Samples failing to meet this threshold were excluded from other time interval classifications. The remaining residual samples were considered to exhibit stable spectral responses and were assumed to remain unchanged throughout the study period.

Before classification, these unaltered samples were randomly divided into a training group (70%) and a test group (30%). This division allowed for the assessment of the accuracy of the generated salinity maps using both the classification algorithm's training and test groups. The process CCDC is as follows: Identification of essential features for salinity determination based on field data; Time series analysis based on the above feature to identify field points whose value does not change over time (280 samples); Time series analysis based on the above feature to generate artificial points whose value does not change over time (576 sample artifacts); Producing a map with points 1 and 2 with different regression methods and comparing their accuracy and choosing the most accurate map from the previous step.

#### Accuracy assessment

Three distinct criteria, RMSE, R^2^, and Mean Absolute Error (MAE), were used to estimate the accuracy of the prepared prediction models (see Supplementary D).

## Results

### Soil salinity estimation using L8 imagery data

#### Feature extraction and segmentation of L8 data

According to Table 2, the results revealed that out of 26 environmental variables (including several auxiliary variables), the "Random Forest-Backward Feature Elimination" (RFE-RF) method^92^. The relative importance of independent variables for estimating soil salinity using L8 imagery is shown in Supplementary Fig. S4. It can be concluded that the parameters obtained from remote sensing are more important than other factors of soil formation, i.e., geomorphology, topography, and EC, in the spatial estimation of soil salinity in the surface horizon.

The MNDWI (0.35%) and NDSI (0.33%) were determined to be important predictors of soil salinity in the RF method (Supplementary Fig. S4a). The NDWI is the third important auxiliary variable, with a relative importance of 0.23%. Moreover, the XGBoost has shown better results in salinity estimation with L8 data. Therefore, the critical factors using L8 data, as shown in Supplementary Fig. S4b, are B3, MNDWI, NDSI, NDWI, NDVI, EVI, MrBVF, TWI, TPI, index S1, and S4.

Furthermore, based on the results presented in Supplementary Fig. S4c, the DT algorithm ranks the relative importance of auxiliary variables as follows: MNDWI, NDSI, NDWI, TWI, SAVI, MrBVF, TPI, NDVI, EVI, S3 (Table 1), in an ascending to descending trend.

#### Accuracy assessments of salinity estimation using L8 data

**Figure 3:**
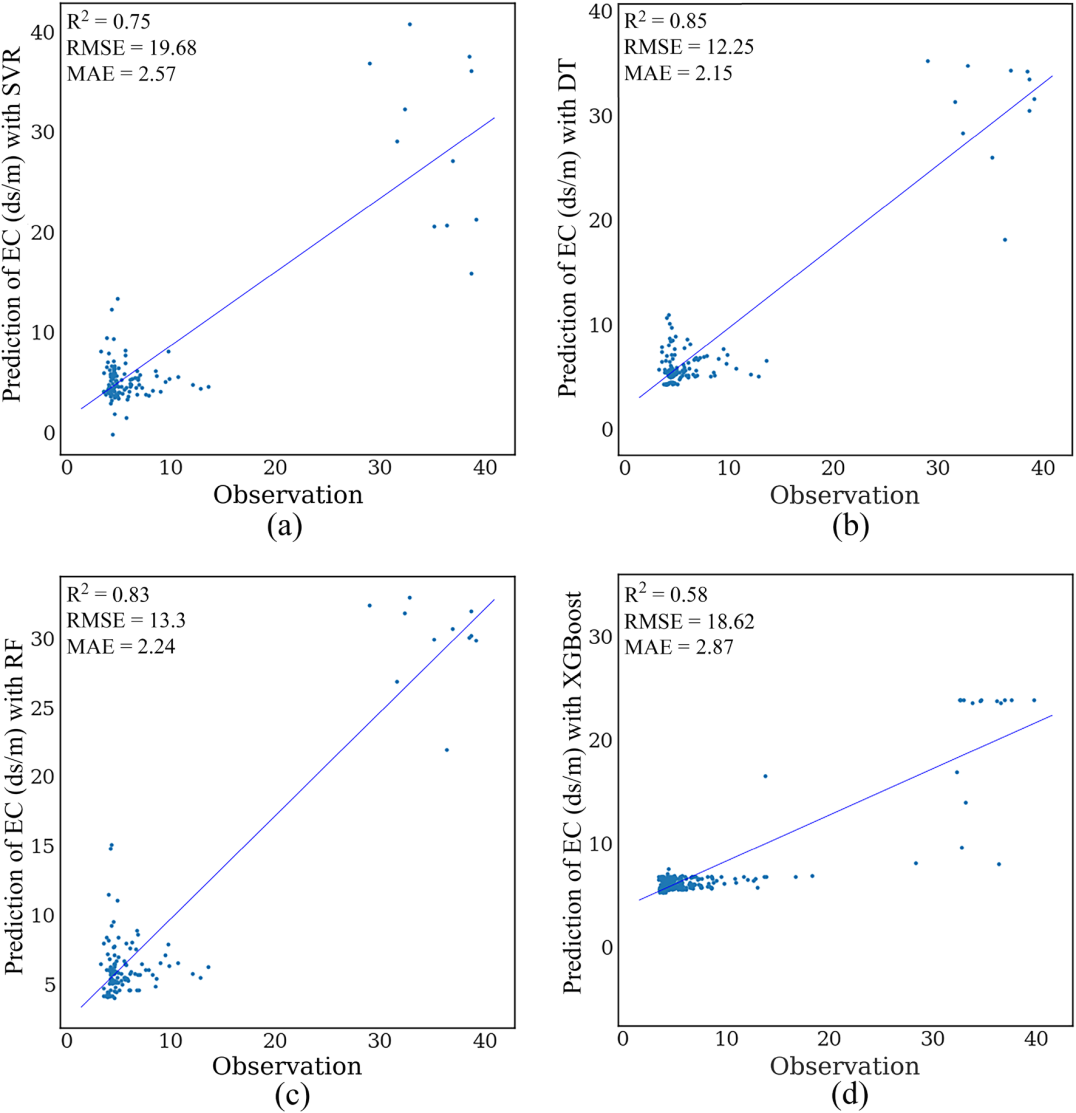
Scatter plots of SVR (**a**), RF (**b**), DT (**c**), and XGBoost (**d**) methods estimating the soil salinity value using the L8 image. The Matplotlib for Python (version 3.7.2) draws graphs^93^.

The scatter diagram of soil EC is shown in Fig. 3. The highest R^2^ value is related to the DT model. Therefore, DT can predict the salinity more accurately than others with RMSE = 12.25, MAE = 2.15, and R^2^ = 0.85. Furthermore, RF has almost the same performance as DT, while the XGBoost algorithm has the lowest coefficient of explanation for soil salinity estimation with RMSE = 18.62, MAE = 2.87, and R^2^ = 0.58).

#### Spatial estimation of Soil salinity with L8 data

**Figure 4:**
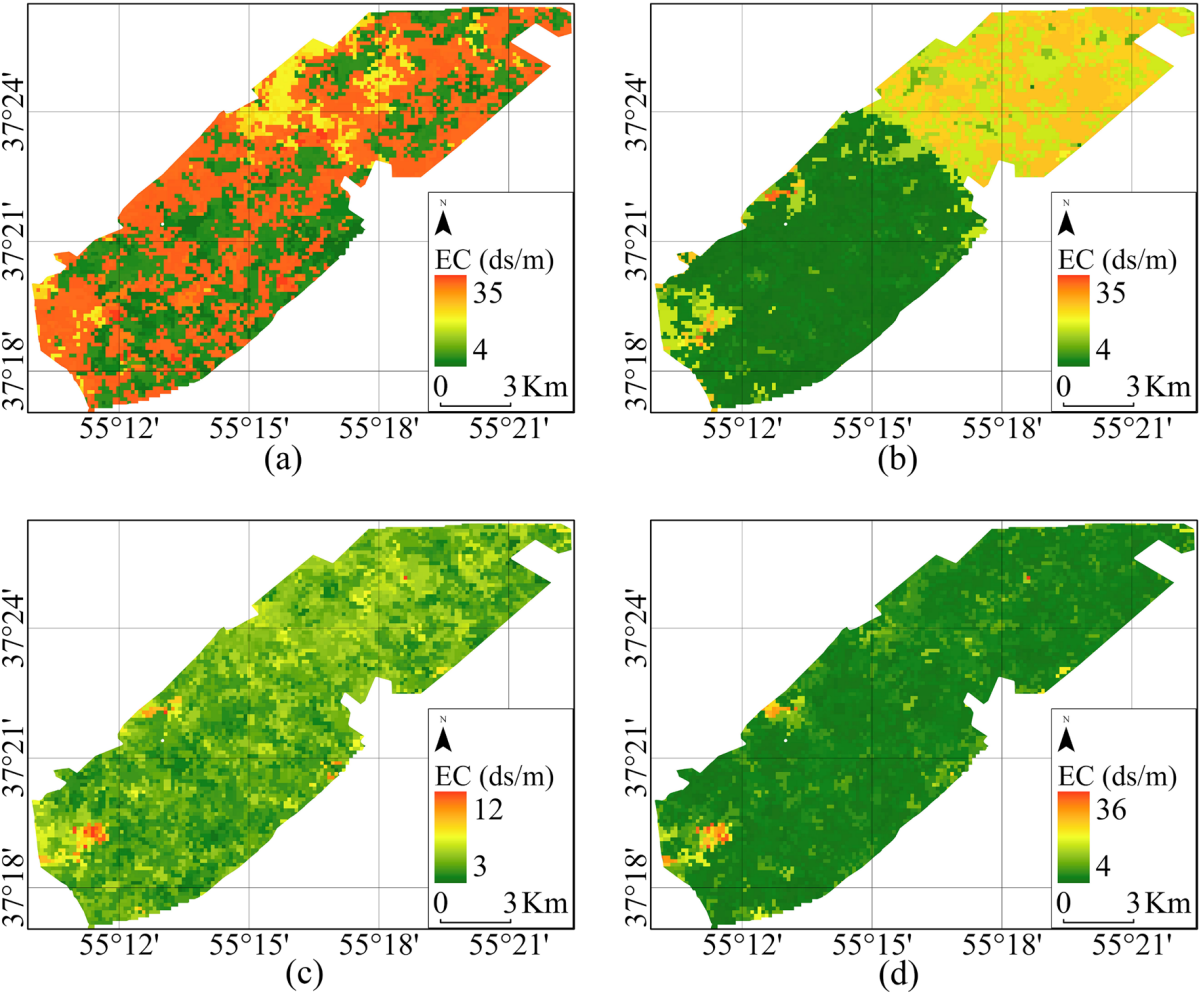
Soil salinity map created by L8 data in 2020 (**a**) XGBoost, (**b**) DT, (**c**) SVR, and (**d**) RF methods.

Suitable bands were determined to train the regression algorithms and create salinity level maps, and pixel data corresponding to sample points within each class were employed as training data. The regression was performed based on five salinity classes, and Fig. 4 displays the results obtained from various supervised regression algorithms.

### Soil salinity estimation using S2 imagery data

#### Feature extraction and segmentation S2 data

As indicated in Table 2, the results revealed that out of a total of 26 environmental variables (including several auxiliary variables), the RFE-RF method^92^ identified the following eight variables as having the highest feature importance: Clay, Carbonate, NDVI, S3, S2, S1, Greens, and Brightness. These variables were derived from band assignments of the L8. Additionally, six parameters, Diffuse, MrVBF, Midslope, Standard, and SAGA, extracted from the DEM^94^, and two climatic parameters, MAP and MAT, were selected for analysis.

Supplementary Figure S5 shows that the importance of environmental auxiliary variables differed in each algorithm using S2 data. The results showed that remote sensing data were the most important predictors of soil salinity. In Supplementary Fig. S5, the essential controllers are auxiliary variables of the humidity index, salinity, modified soil index, MrVBF, and climate variables of MAT, NDVI, S3, and TWI (Table 1). Soil salinity models with advanced DT algorithms have the highest effect on the prediction of soil salinity in the study area. This shows that humidity, altitude, elevation, and vegetation are the most important factors of soil formation in the study area. It is effective in the spatial distribution of soil and its characteristics because the topography and vegetation of each region are some of the essential and influential characteristics of the soil characteristics of that region, including soil salinity.

#### Accuracy assessments of salinity estimation using S2 data

**Figure 5:**
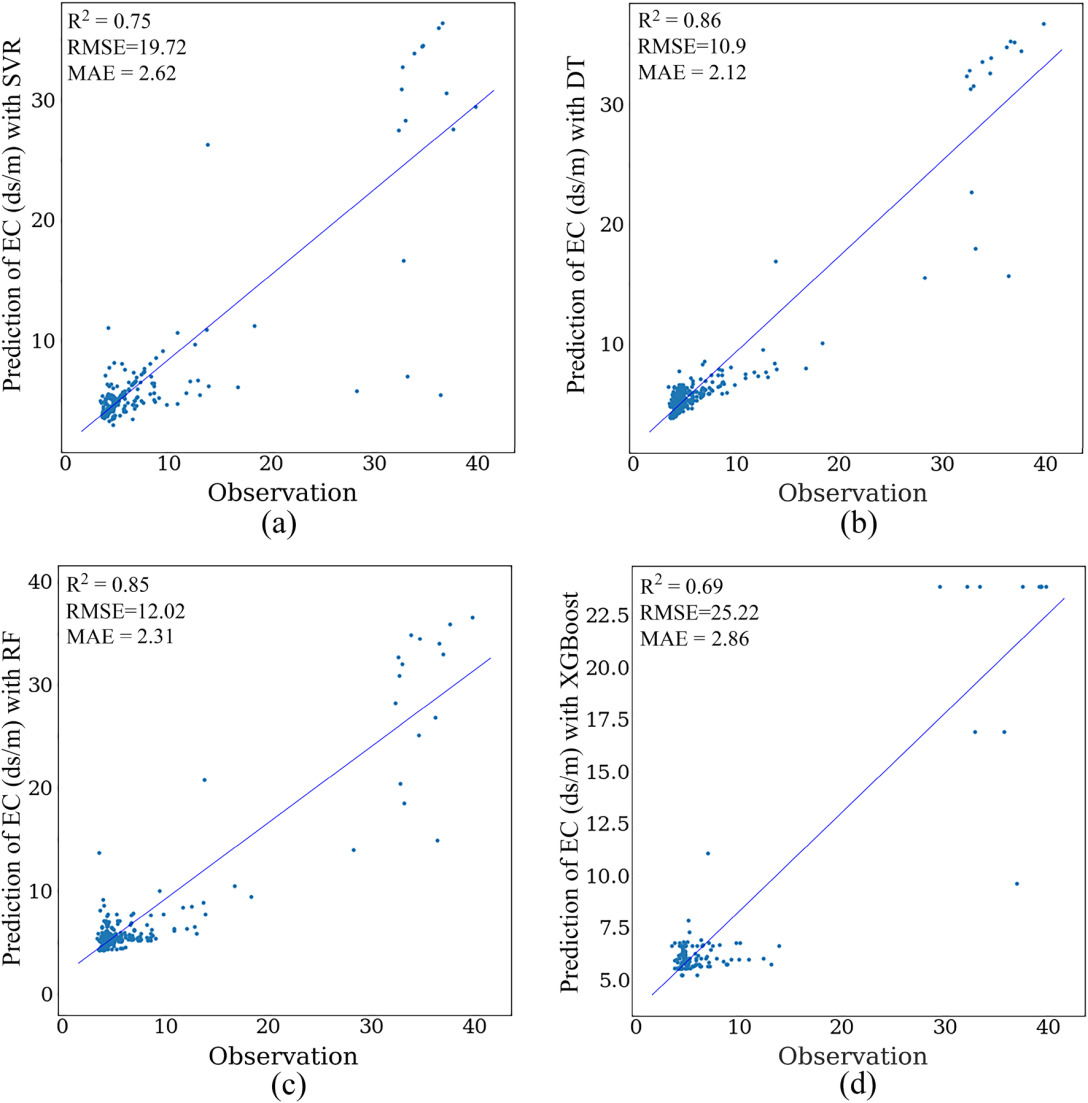
Scatter plots of SVR (**a**), RF (**b**), DT (**c**), and XGBoost (**d**) methods estimating the soil salinity value using the S2 data. The Matplotlib for Python (version 3.7.2) is used to draw graphs^93^.

As seen in Fig. 5, the DT had the highest value of R^2^ and was the best model for estimating EC. The best-fitted model with the highest explanation coefficient and the lowest error was selected. Based on the results obtained from the estimator’s machine learning, the SVR has the lowest accuracy, and the DT method has the most accuracy.

#### Spatial estimation of Soil salinity with S2 data

**Figure 6:**
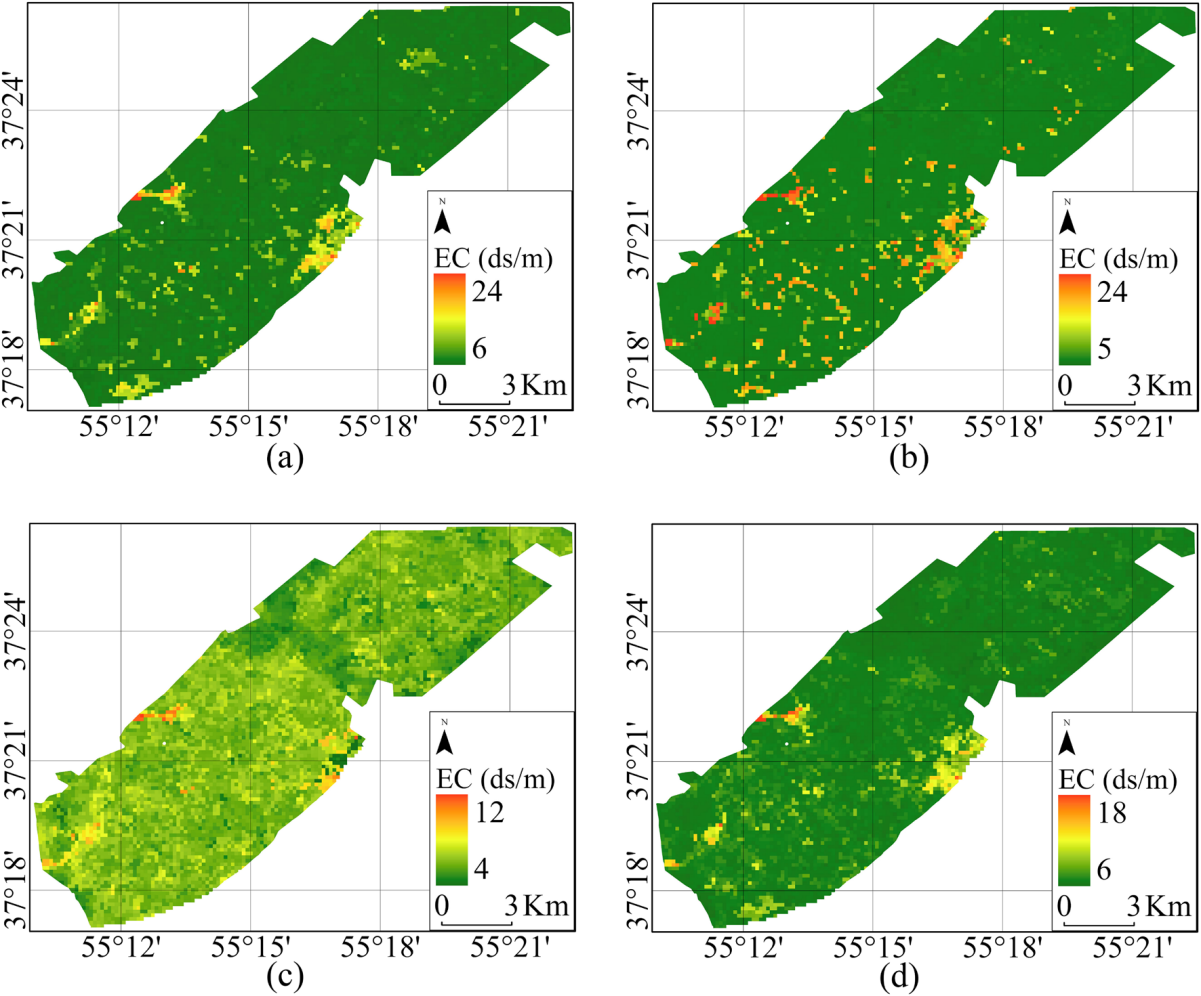
Soil Salinity map created with S2 data in 2020 using (**a**) RF, (**b**) XGBoost, (**c**) SVR, and (**d**) DT.

Figure 6 illustrates the classified salinity map of the study area, created using S2 data. It highlights the effectiveness of drainage in mitigating soil salinity. The RF algorithm exhibited higher accuracy than others due to its utilization of more trees and optimal bootstrap sampling techniques for auxiliary variables and observation points. The DT algorithm also performed well, while the SVR demonstrated improved accuracy over the XGBoost model.

The map indicates a range of soil salinity values in the study area, with the highest recorded at 24 ds/m and the lowest at 4 ds/m. These areas primarily consist of agricultural fields with low slopes and are equipped with pipe drainage systems.

#### Temporal analysis of soil salinity using L8 imagery data

We conducted temporal analysis on soil salinity exclusively using L8 imagery data. Supplementary Figures S6, S7, and S8 show the soil salinity map generated using RF and L8 data between 2013 and 2020. They revealed a decreasing trend in salinity levels in the study area. The Golestan drainage area started in 2013 and ended in 2019, and the Bostan drainage area began in 2019 and was completed in 2023. This can be attributed to the extensive use of pipe drainage in agricultural regions and potential climate changes. Also, the average soil salinity time series ten years after installing the drainage network is shown in Supplementary Figs. S9 and S10. In 2012, the predominant and least prevalent land classifications were those subject to high salinity restrictions (54%) and those free of salinity restrictions (2%), respectively. However, by 2023, the most prevalent land classification had shifted to areas without salinity limitations (40%), while those with very high salinity restrictions constituted 7% of the region's landscape.

Over the period from 2012 to 2023, the extent of land subject to high salinity restrictions experienced a significant decrease in drainage areas, nearing parity with the extent of land free of salinity restrictions. This transformation suggests a notable reduction in salinity levels within the drainage area and an overall increase in salinity across other non-drained regions.

Upon analyzing the histograms for 2012, it's apparent that salinity in agricultural regions primarily falls within the range of 8–18, with a peak around 13 (Supplementary Fig. S11). In the 2023 histogram shown in Supplementary Fig. S12, the minimum and maximum values have decreased, now confined to a range of 4–15, indicating an overall reduction in salinity in agricultural areas. Notably, the shift in the peak of the histogram to the left suggests that regions with higher initial salinity values within this range have experienced more pronounced reductions, indicating a significant improvement in their salinity conditions.

A 7-year analysis of soil salinity spanning from 2013 to 2020 was conducted using Landsat 8 (L8) imagery, focusing on the differentiation of pixels between bare soil and plant cover. This analysis aimed to identify optimal indices and suitable timing for studying changes. A comparison of the soil salinity maps for 2013 and 2020, illustrated in Supplementary Fig. S13, indicates a decreasing trend in salinity, particularly noticeable in agricultural areas with tile drainage systems. However, rangeland areas display a notable trend potentially influenced by climate change.

A slight increase is observed within the range of high salinity levels when comparing data from 2013 to 2020, consistent with global trends. Consequently, salinity has risen in non-agricultural regions but declined in agricultural areas. A distribution chart comparing values from 2013 has been generated for further investigation. The histograms and this distribution chart confirm that changes diminish within the 8–18 salinity range, displaying a negative regression slope. This indicates that areas with initially higher salinity levels experienced more significant reductions. However, in cases exceeding a salinity value of 18, a relative increase in salinity is observed. On average, this increase amounts to approximately five units of EC for values greater than 18 (Supplementary Fig. S14).

**Figure 7:**
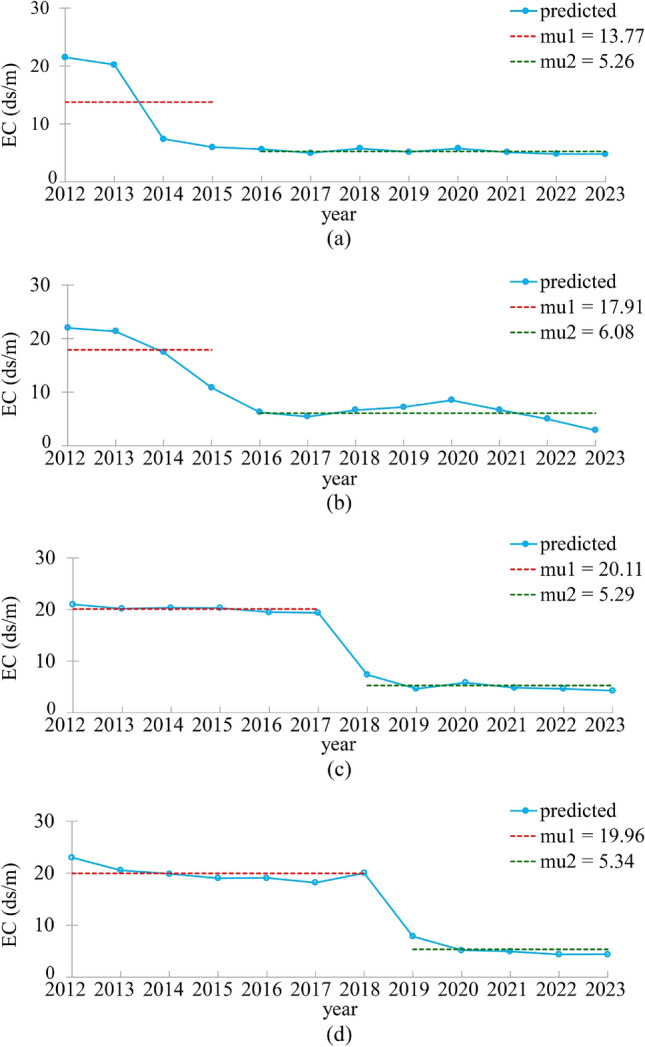
Trend diagram of Kendall and Pettit Mutation test for phases 1, 2, 3, and 4 of drained areas.

Figure 7 compares soil salinity and provides the slope (β) and significance level (P) values related to the salinity trend in drained areas. Based on these values, it can be concluded that soil salinity has decreased with drainage. Drained regions exhibit a significant decreasing trend at a 5% level, with the most substantial decrease observed in 2013. Based on Kendall's statistics, the analysis of soil salinity changes in the study area over the 11 years (2012–2023) indicates a consistent downward trend in salinity changes following drainage implementation. However, the situation is different for non-drained areas.

## Discussion

### Estimation of soil salinity using S2 and L8 data

The study compares four machine learning methods, RF, DT, XGBoost, and SVR, to estimate soil salinity levels using remotely sensed L8 and S2 data. We have found that the accuracy of DT and RF in salinity estimation at a depth of 10 cm is higher than XGBoost and SVR. This shows that RF and DT are more powerful in modeling highly nonlinear dimensional relationships than XGBoost and SVR. Our findings concur with those found in^95,96^. Merembayev et al.^97^ indicated that DT and RF have almost the same performance for soil salinity estimation, which is aligned with our findings. Ding et al.^98^ used the Landsat Enhanced Thematic Mapper Plus (ETM +) image, stating that DT is suitable for extracting saline soil information. Qi-sheng et al.^99^ found the effectiveness of DT for soil salinity extraction from ETM data, which is aligned with our findings. Fu et al.^100^ reported that a combination of DT using Landsat TM images had superior performance in land salinization classification. In addition, Breiman^80^ stated that by aggregating multiple models, the instability of a single-tree model is minimized, which leads to an improvement in consistency^101^. While our results demonstrate the superiority of DT over RF for indices developed by L8 imagery data, Haq et al.^102^ have argued that RF outperforms DT using L8 imagery data. It is worth noting that their soil samples were collected at a depth of 15 cm, whereas our study samples were collected at a depth of 10 cm. Nevertheless, our findings indicate a close alignment between the predicted and actual EC values when using the RF and DT models. This contrasts several studies showing RF superior to DT for estimating soil salinity^103–105^.

### Feature importance for estimation of soil salinity

Our study shows the advantage of ranking the relative importance of predictor variables (indices) using machine learning methods. Salcedo et al.^106^ have stated that applying different spectral indices helps characterize soil salinity. Accordingly, our results suggest that MNDWI is an optimal index for assessing soil salinity using the DT and RF methods with both L8 and S2 imagery data. This is aligned with Xu^46^, Qi-sheng et al.^99^, and Fu et al.^100^, stating that MNDWI is suitable for salinization mapping. Also, Ding et al.^98^ reported the usefulness of MNDWI combined with NDVI in soil salinity classification. Additionally, our results indicated that the NDSI is the optimal index for salinity estimation using S2 data. Aligning with our findings, Shrestha et al.^107^ have found that the only significant predictor of observed soil salinity was NDSI.

According to our study, topography and vegetation indices are important indicators of soil salinity. This is aligned with the findings of Wang et al.^108^. They indicated that environmental factors contributed substantially to soil salinity estimation, including the digital elevation model (DEM) and Green Atmospherically Resistant Vegetation Index (GARI).

Additionally, our findings show that the salinity index reaches its maximum value, particularly near the soil surface in specific sections. Factors such as capillary currents, soil texture, groundwater, and moisture levels are pivotal in transporting solutes to surface layers, especially during dry seasons. Consistent with our findings, Taghizadeh-Mehrjardi et al.^55^ identified climate parameters, particularly prolonged droughts, as the primary factors influencing soil salinity in lowland areas. They also highlighted saline parent materials, soil texture, and the lack of surface irrigation and drainage as contributing factors.

Moreover, our findings showed that in areas with saline soil, an increase in soil moisture leads to a decrease in reflectance within the visible and near-infrared regions, resulting in enhanced soil salinity estimation, as also reported by Cao et al.^109^.

### Temporal and spatial changes in soil salinity

Analyzing soil salinity changes from 2012 to 2023 in the study area indicates that most of these lands did not transition from saline to non-saline over 11 years in the undrained areas. The extent of moderate and high salinity classes gradually decreased, while the area of lands with very high and severe salinity increased. Despite the similar influence of climate and weather changes on salinity increase, agricultural areas with drainage systems have experienced reduced salinity. Additionally, areas with improved soil amendments and established drainage systems exhibit more favorable salinity classifications than non-drained areas^110^. Moreover, regions with drainage systems in place report higher crop yields than non-drained areas; this is corroborated by the soil salinity chart in this range, which demonstrates a declining trend, affirming the effective functioning of drainage systems. Additionally, the high EC of drainage water, signifying a very high salinity class, indicates that drainage systems have efficiently removed salinity from the soil, and these changes have stabilized over time^110^. This is aligned with Gopalakrishnan et al.^111^, as they reported that poor drainage intensifies soil salinity, and salinization has severe implications for food production and security. Therefore, this indicates the importance of installing a proper drainage system aligned with Singh's findings^112^.

Since the soil salinity of the grazing lands around the outlet drainage evaporation pond has gradually increased over the 11 years following the implementation of the pipe drainage project, it is recommended to consider a comprehensive Integrated Water Resources Management (IWRM) plan. This plan would utilize serial biological drainage for agricultural drainage waters multiple times, each cycle devoted to cultivating salt-tolerant crops. The highly saline drainage water produced at the end of this process can be repurposed for non-agricultural activities such as aquaculture or salt production. Eventually, a small volume of water should be directed into the outlet drawing, or ideally, no water should enter the outlet and instead be discharged directly into the sea. Mardanifar et al.^113^ investigated the declining salinity trend in the Golestan and Boustan dam areas. Their findings regarding the trend of electrical conductivity (EC) changes in the sampled wells within the project area generally indicate a reduction during the operation of the drainage system. Factors such as the terrain slope, positioning of drainage systems, and the known distribution of soil salinity are considered significant in this region.

Overall, proper drainage is an essential aspect of managing soil salinity. It helps to remove excess salts from the soil and prevent their accumulation. Drainage systems such as tile drains or subsurface drainage can be implemented to improve water movement and reduce salt build-up in the root zone. Effective drainage can help to lower the water table, preventing the capillary rise of saline groundwater. It also promotes leaching, which involves flushing salts from the soil profile through water movement. This helps to maintain a more favorable salt balance in the soil, reducing the risk of salinity problems. Farmers can effectively manage soil salinity by implementing proper drainage measures and creating a better plant-growing environment. This can improve yields, improve water-use efficiency, and enhance soil fertility. In addition, proper drainage can help minimize the environmental impact of soil salinity. It reduces the risk of saltwater intrusion into freshwater sources, protecting water quality and preserving aquatic ecosystems. Overall, drainage plays a crucial role in soil salinity by preventing salt accumulation, improving water movement, and promoting optimal plant growth and agricultural productivity.

### Limitations

The assessment of soil salinity change trends through the utilization of remote sensing data has a few limitations. The principal constraints in this study are as follows: (1) Remote sensing data is typically collected based on a specific scale, with each data pixel potentially representing several meters. This spatial scale may result in losing fine details related to soil salinity changes at smaller scales. (2) Acquiring remote sensing data over time and for specific areas can be challenging. Some regions may have limited access to satellite imagery, which can restrict the comprehensive evaluation of soil salinity changes. (3) Cloud cover in satellite imagery can introduce interferences in the remote sensing process and degrade image quality. Accurate soil salinity monitoring requires high-quality, cloud-free images.

## Conclusion

The study utilized remote sensing imagery data and soil salinity samples in machine learning algorithms to explore factors affecting salinity levels in salt-affected crop fields. The DT machine learning model effectively predicted total soil salinity in heavily vegetated croplands. The study highlighted that undrained areas exhibit greater sensitivity to salinity, likely influenced by climate patterns. Furthermore, a significant disparity in average salinity levels between undrained and drained land was observed, with lower EC in agricultural land due to salt leaching. To mitigate salinity, the study recommends the installation of drainage pipes at a depth of 2 m to reduce and stabilize soil electrical conductivity. The results suggest incorporating environmental variables in time series modeling to predict soil salinity, considering that climate and weather variations contribute to increased salinity.

### Supplementary Information

Supplementary Information.

## Data Availability

According to the first author's university regulations, the datasets used and/or analyzed during the current study are available from the corresponding author upon reasonable request.
