# Supplementary material for: A longitudinal analysis of soil salinity changes using remotely sensed imageries
Source: Sci Rep. 2024 May 6;14:10383. doi: 10.1038/s41598-024-60033-6 (PMC11074301; doi:10.1038/s41598-024-60033-6)
Supplement: Supplementary file 1 — Supplementary Information. [file 41598_2024_60033_MOESM1_ESM.docx]

**Supplementary information**

## Supplementary A. Feature selection and segmentation

The segmentation process precedes the feature extraction to reduce noise impact. Instead of pixel-based calculations, indices are computed within each image segment. Various parameters such as scale, shape, color, and compactness are employed, with their relative weighting importance considered, to create image segments from satellite imagery. This research utilized multi-resolution segmentation methods to capture natural variations. Achieving a balance between segment indices and landscapes' spectral and structural characteristics is critical. Proper indices for multi-resolution segmentation are selected through trial and error, involving multiple iterations with different indices and weight combinations. Regarding spatial conformity with landscape features, segmentation accuracy significantly influences the final precision of image classification and identification.

Optimal segmentation levels were determined based on spectral and spatial resolution, using a trial-and-error approach at various levels. This involved conducting multiple segmentations using S2 and L8 satellite images, along with numerical contour maps, slope, geological, and soil base maps as primary layers, all geocoded and processed iteratively to identify the most effective combination of segmentation parameters such as scale, shape, and compactness for soil salinity assessment. Images were then processed using the Simple Non-Iterative Clustering (SNIC) algorithm for segmentation. The SNIC method evenly distributes seeds throughout the image to create super-pixels. A priority queue determines the next pixel assigned to a cluster based on distance from its centroid. This process continues until centroid convergence. In the classification phase, quantitative analysis is conducted to specify the desired landscape characteristics and define thresholds for isolating individual segments from their surroundings. An algorithm is selected, and coding is implemented in Google Colab for segment classification. Object-oriented techniques are employed to classify segments associated with soil samples using a hierarchical series of codes in Google Colab.

Within the Google Earth Engine environment, satellite images and base maps are utilized with Google Colab. Initial training soil samples are identified within the segmented images, and various indices related to each object (about training samples) are analyzed to select the most suitable indices. Classification is then performed using each index's chosen indices and predefined thresholds. Finally, test samples are employed to assess the accuracy of each index for regression analysis and the introduction of a semi-automated modeling approach for evaluating soil salinity.


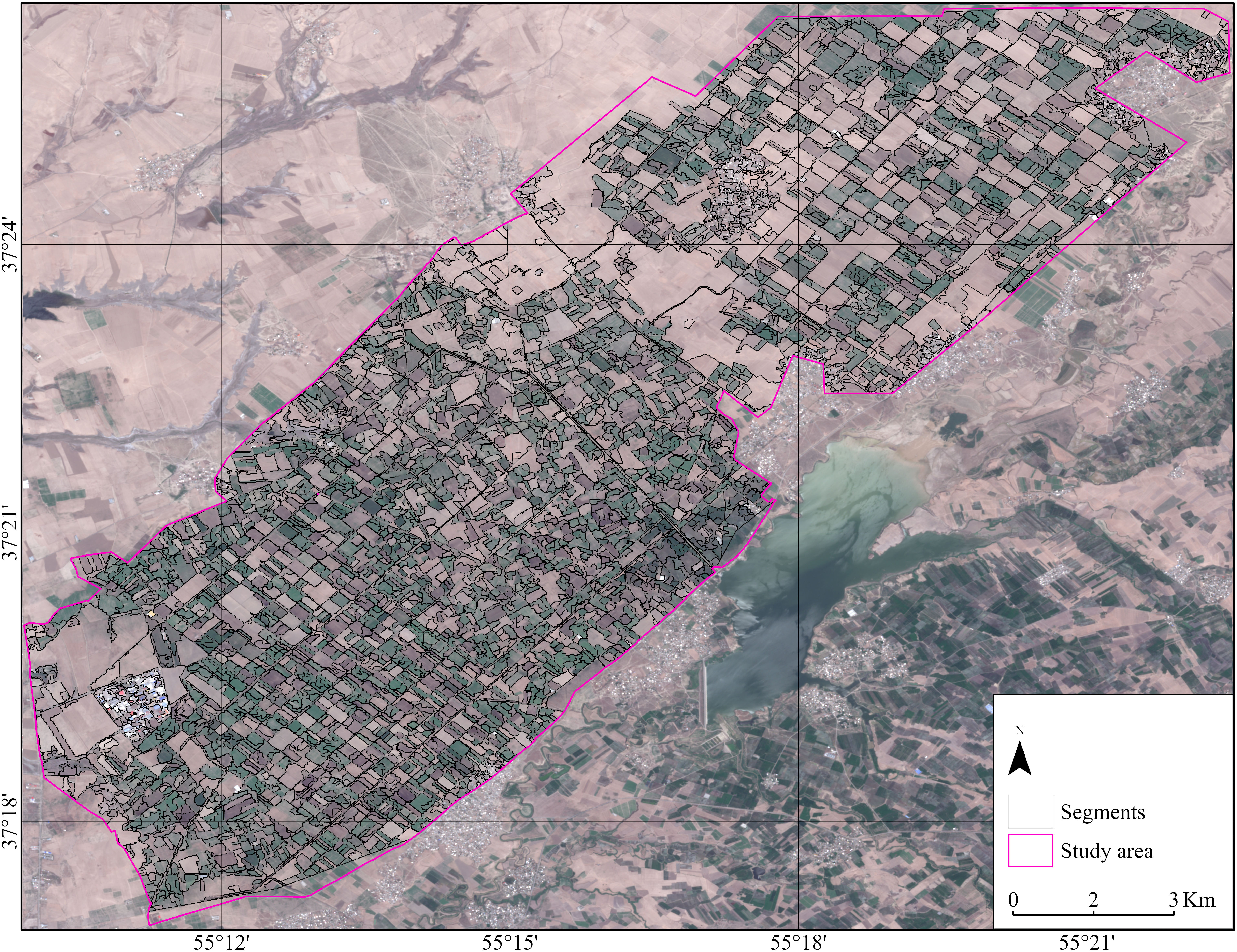


**Figure S1.** Illustration of the segmented image of the study area.

## Supplementary B. Hyperparameters

***DT Hyperparameters***

The Bayesian optimization from DT regression modeling hyperparameters is shown in Table S3. Hyperparameters, with the best optimum value obtained with regression max feature = "auto, n_estimator = 200, max depth =6, min_samples_split = 4, without bootstrap, random_state= 8 and learning rate= 0.02 have been tested (Table B3).

**Table S1.** DT regression modeling hyperparameters from the Bayesian optimization.

| Parameters | Range | Optimum Value |
| --- | --- | --- |
| n_estimators | 150 | 200 |
| Max feature | [Auto, Sqrt, Log2] | auto |
| Max depth | 1 to 10 | 6 |
| min_samples_split | [2,4,8] | 4 |
| Bootstrap  Random _state  Learning rate | [True, False]  0-50  **0.01-1** | False  8  0.02 |

***RF Hyperparameters***

The RF incorporates key hyperparameters, including *max_depth*, *max_samples_leaf,* *min_sample_split*, *n_estimators*, and *max_features*. The *max_depth* defines the maximum depth of a tree in the RF. The *min_sample_split* determines the minimum number of observations required in each node before it can be split. Its acceptable range spans from 2 to 8. The *min_sample_leaf* specifies the minimum number of samples that should be present in a leaf node. Its range is typically between 4 and 10. The *n_estimators* is the number of estimators in an RF model, or this hyperparameter controls the number of DTs. The *max_features* affects how each tree in the RF is constructed by controlling the maximum number of features considered at each split. These tree-specific criteria significantly affect the model's performance [99].

**Table S2**. Bayesian search cv by hyperparameters for RF

| parameters | range | Optimum Value |
| --- | --- | --- |
| n_estimators | 100 to 200 | 115 |
| Max feature | [none, sqrt, log2] | 'sqrt' |
| Max depth | 1to 10 | 8 |
| min_samples_split  min_sample-leaf | [2,8]  [5,10] | 6  6 |
| bootstrap | [True, False] | false |
| criterion | [squared error, absolute_error] | Squared error |


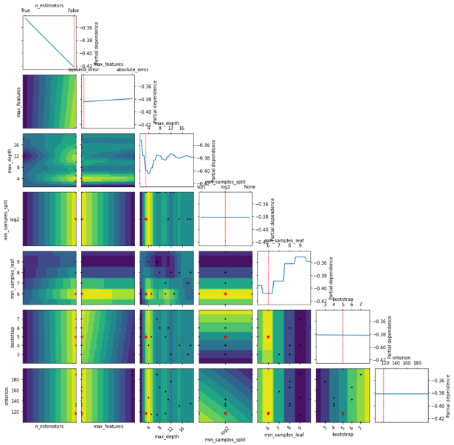


**Figure S2**. Illustrating hyperparameter Bayesian search cv with RF

***SVR Hyperparameters***

In SVR, one essential hyperparameter is C, determining the strength of regularization [82]. Notably, regularization strength is inversely proportional to C. The C has to be positive. In this context, the typical range for C falls between 500 and 750 [83]. SVR also offers various kernel options, including polynomial, Radial Basis Functions (RBF), and sigmoid functions. The RBF kernel will be employed if no kernel is explicitly specified by default. Additionally, if a callable function is provided, it can be used to pre-compute the kernel matrix from data matrices. This matrix should have specific shape requirements for compatibility with the SVR algorithm [71].


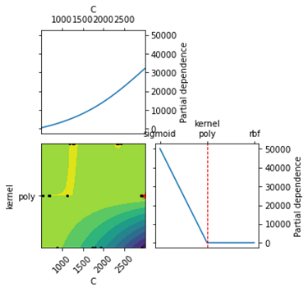


**Figure S3.** Illustrating Hyperparameter Bayesian search cv with SVR

***XGBoost Hyperparameters***

Similar to RF, XGBoost's algorithm is tuned using multiple hyperparameters. A Bayesian optimization on hyperparameters with 10-fold cross-validation was implemented to discover the great model according to R^2^ metrics (Table S2).

**Table S3.** The Bayesian optimization XGBoost regression modeling hyperparameters.

| Parameters | Range | Optimum value |
| --- | --- | --- |
| n_estimators | 70 to 500 | 400 |
| Max depth | 1 to10 | 6 |
| gamma | 0.1-1 | 0.9 |
| reg_alpha  reg_lambda  Learning rate | 1-0.1  1-10  0.01-1 | 0.8  8  0.2 |

## Supplementary C. Penalty computation

Penalty norm:

- 'None': No penalty is added.

- 'l2': An L2 penalty term is added (default choice).

- 'l1': An L1 penalty term is added.

- 'elastic net': L1 and L2 penalty terms are added.

Solver: The solver refers to the method used to solve the optimization problem. The default solver is 'lbfgs.' 'Liblinear' suits small datasets, while 'Sag' and 'Saga' are faster for larger datasets.

“Tol”: 'Tol' is used as a stopping criterion during the optimization process.

“C”: ‘C’ should be a positive float value and is the inverse of the regularization strength. Smaller 'C' values indicate more robust regularization, similar to how it works in SVR.

“Fit Intercept”: This hyperparameter determines whether a constant term should be added to the decision function.

Understanding and fine-tuning these hyperparameters is crucial for optimizing the Maximum Entropy model for specific tasks and datasets.

## Supplementary D. Accuracy Assessment

| $R^{2}=1- \frac{{\sum_{i=1}^{n} \left( Q_{i}-p_{i} \right)}^{2}}{{\sum_{i=1}^{n} \left( Qi-\bar{Q} \right)}^{2}}$ | (1) |
| --- | --- |
| $RMSE=\sqrt{{\frac{1}{n}\sum_{i=1}^{n} \left( Q_{i}-p_{i} \right)}^{2}}$ | (2) |
| $MAE=\frac{1}{n}\sum_{i=1}^{n} \left\vert Q_{i}-p_{i} \right\vert$ | (3) |

where *Q_i_* indicates a true valve, and *p_i_* is a predicted value. A Ǭ is the mean of the observed data and total data values.

## Supplementary E. Figures and tables





**Figure S4.** Feature importance in (a) RF, (b) XGBoost, and (c) DT using L8 data





**Figure S5.** Feature importance in (a) RF, (b) XGBoost, and (c) DT using S2 data

## Supplementary F. Temporal analysis


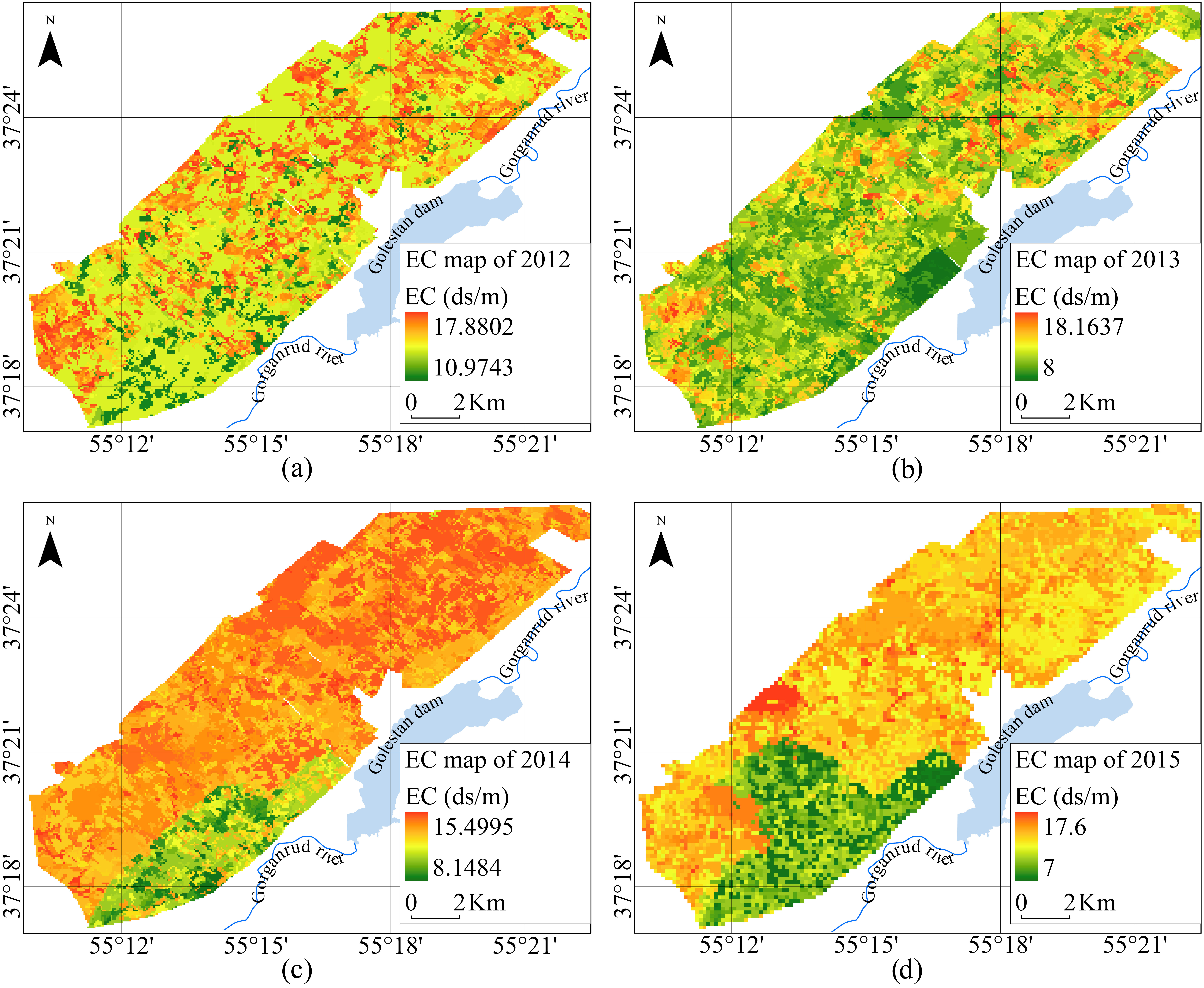


**Figure S6.** The map of soil salinity changes from 2012 to 2015 in drained and undrained areas with the L8 and RF algorithms.


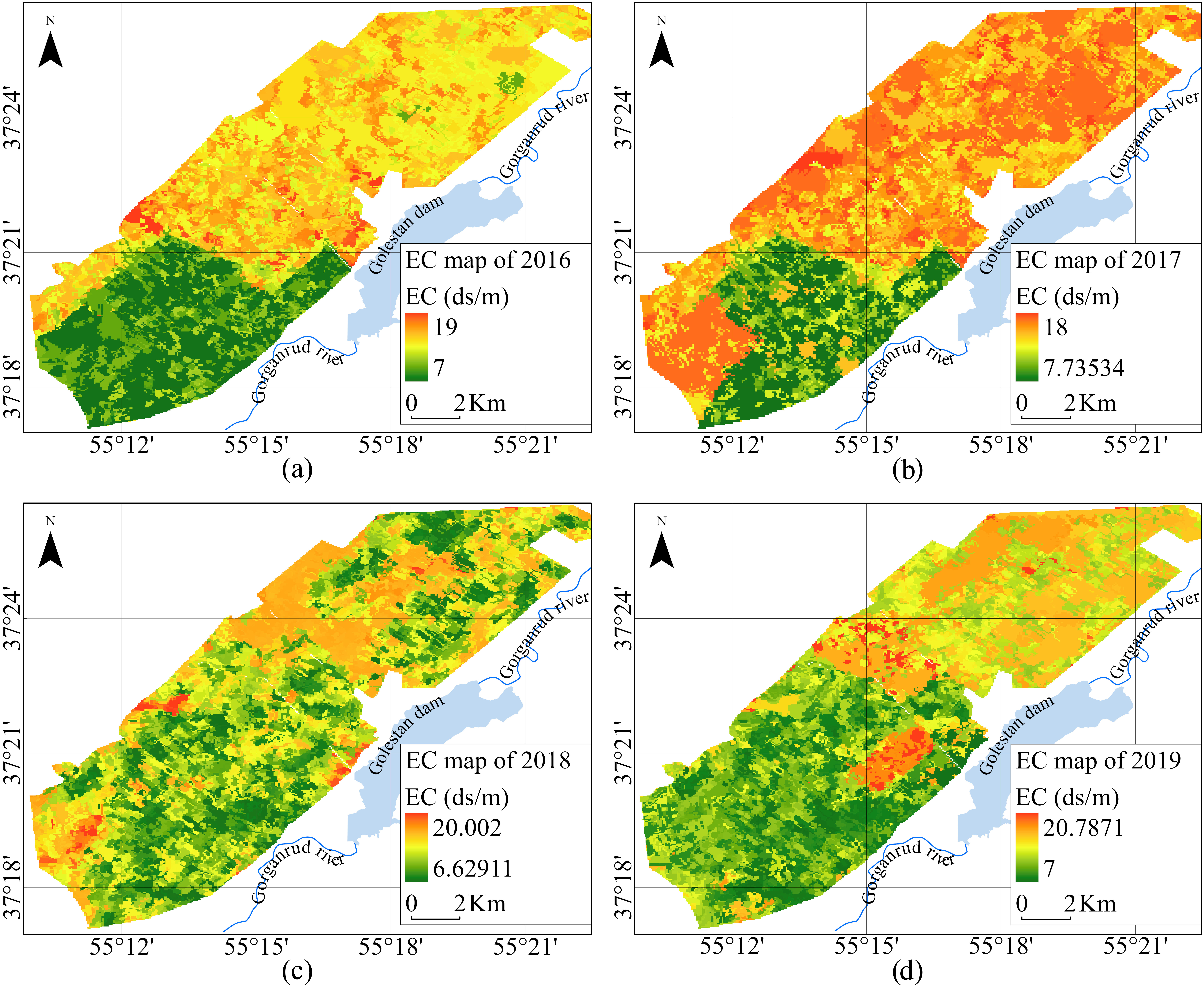


**Figure S7.** The soil salinity map changes from 2016 to 2018 in drained and undrained areas with the L8 and RF algorithms.


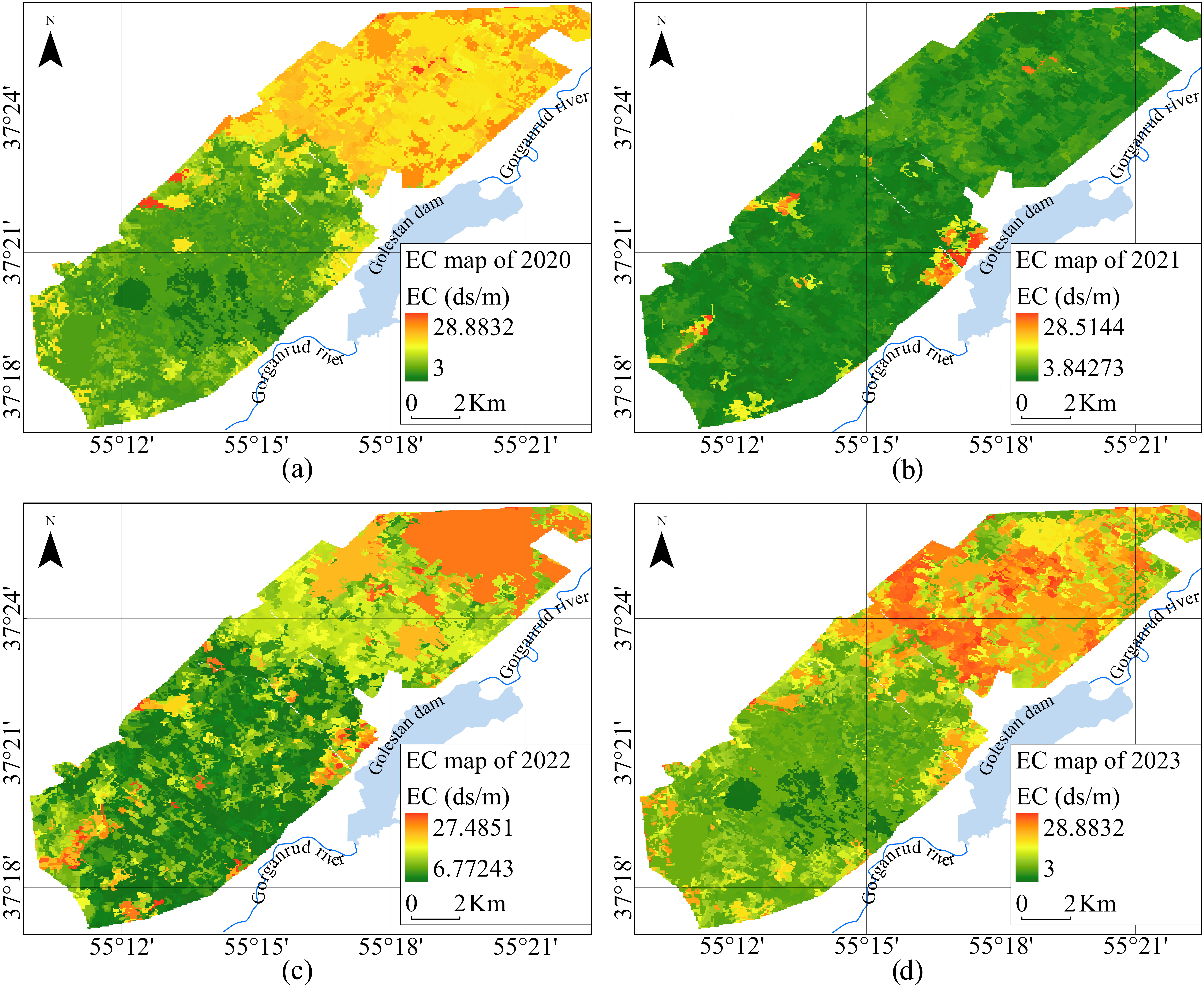


**Figure S8.** The soil salinity map changes from 2020 to 2023 in drained and undrained areas with the L8 and RF algorithms.


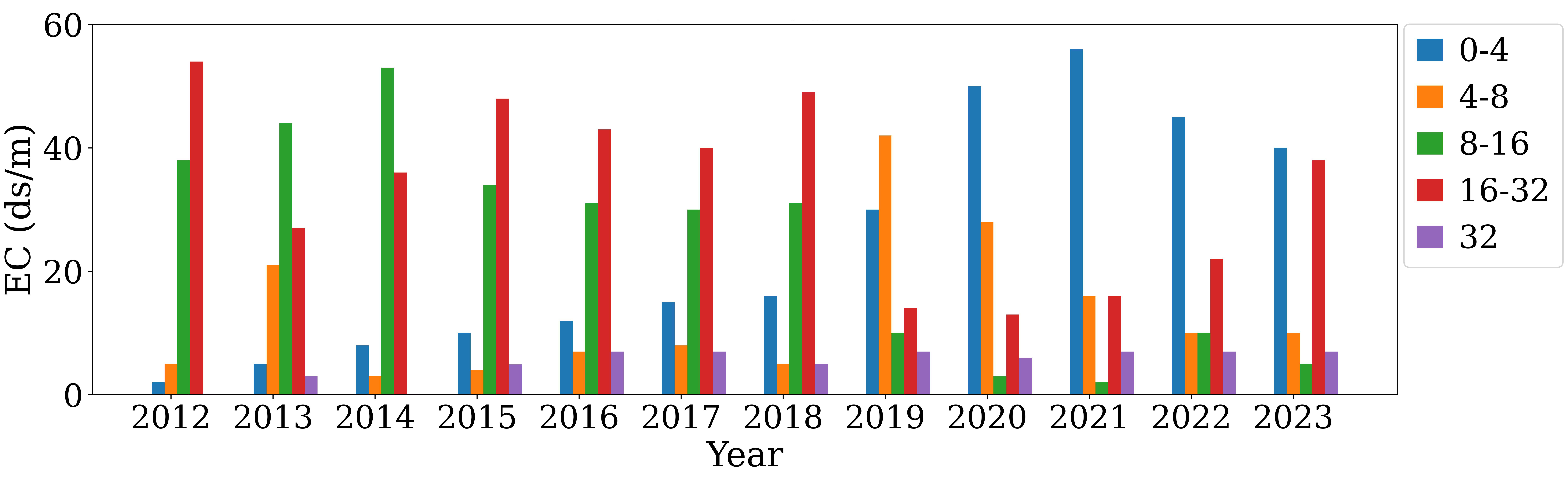


**Figure S9**. Graph of percentage sample with different classes of (A) EC from 2012 to 2023. EC classes are defined as: <4: very low; 4–8: low; 8–12: moderate; 12–16: high; 16–32: very high; >32:

extremely high.


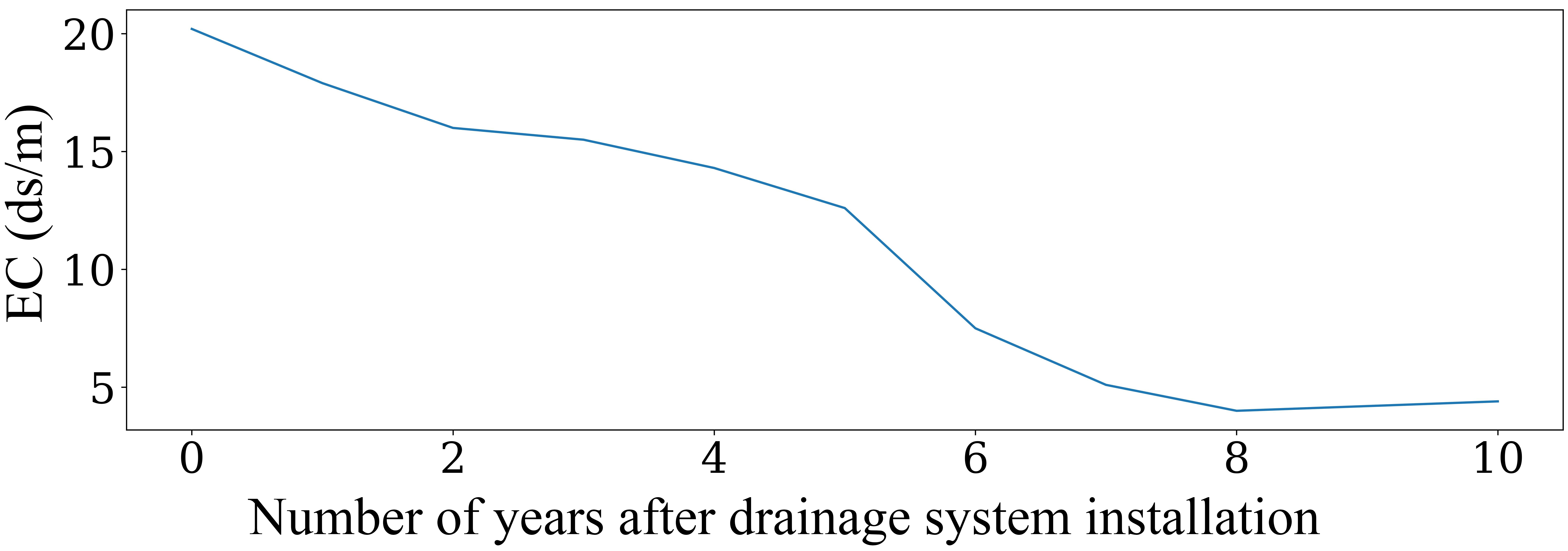


**Figure S10.** Average EC in ten years after installing drainage network in the study area


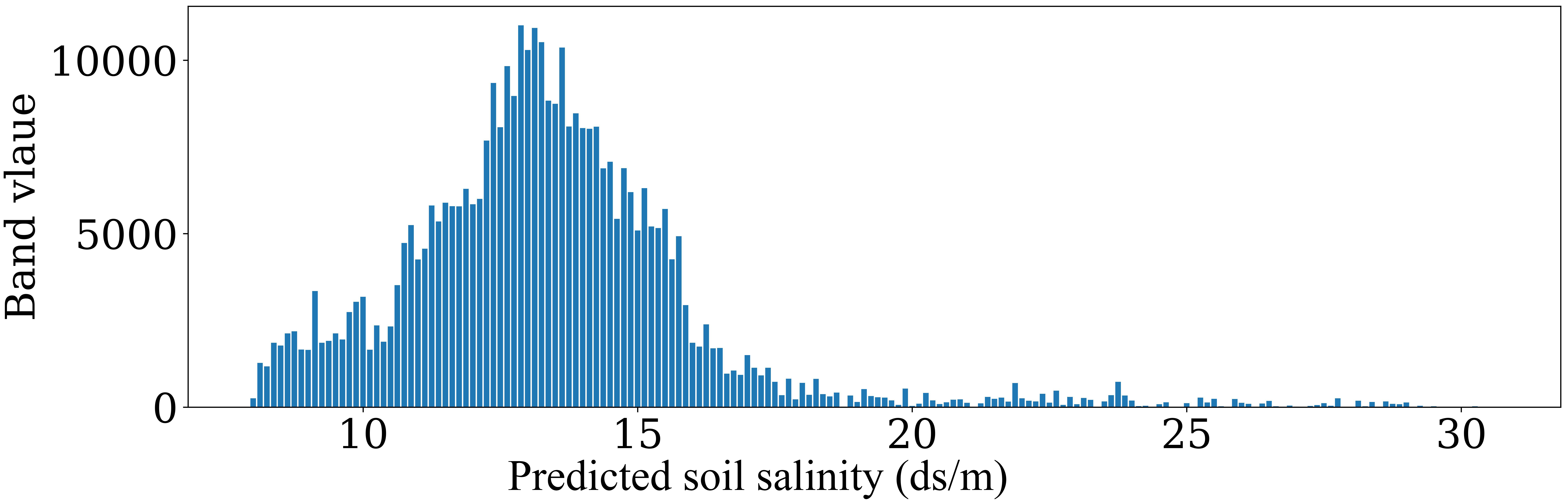


**Figure S11.** Histogram of changes in the salinity content in 2012


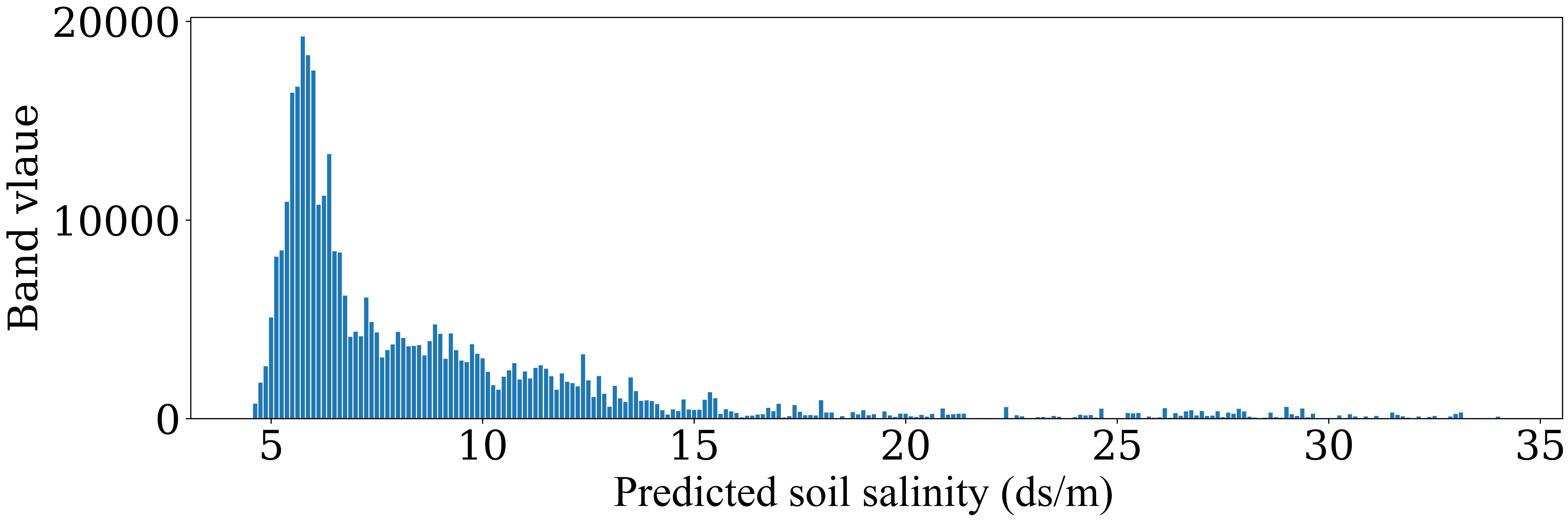


**Figure S12.** Histogram of changes in the salinity content in 2023


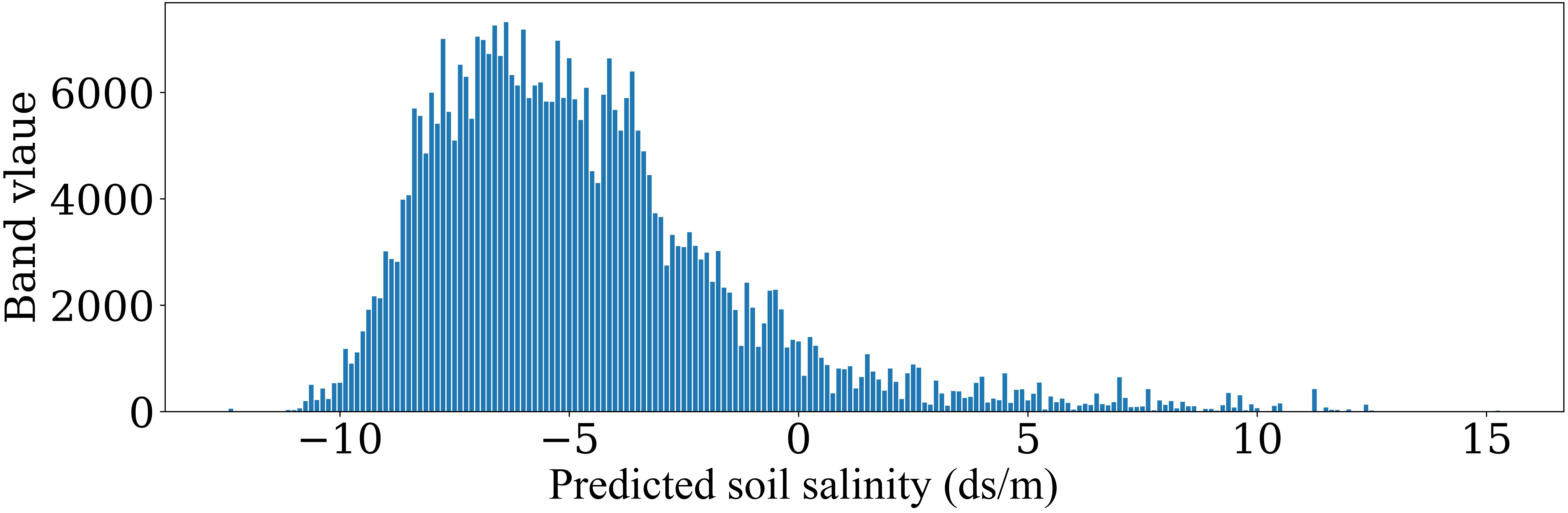


**Figure S13.** Histogram of difference of change in salinity content from 2012 to 2023


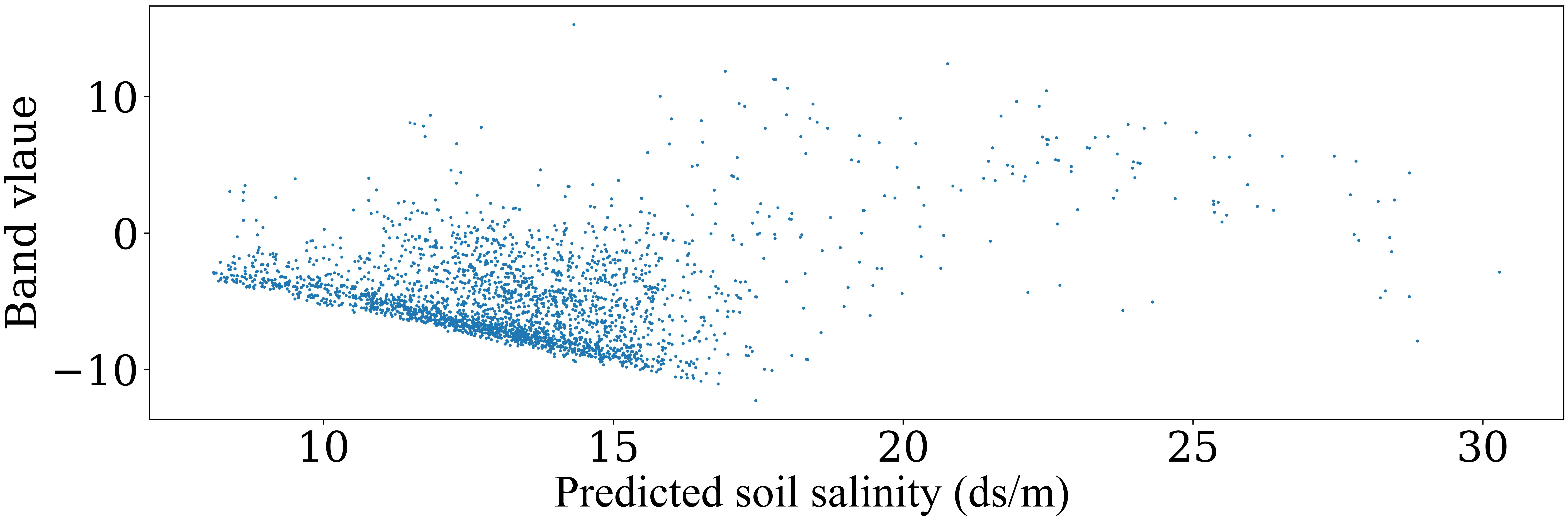


**Figure S14.**  Scatter distribution of changes of 2023 compared to 2012
